# ‘Doing it together?’ A dyadic qualitative study of perceptions, facilitators, barriers and expectations for physical activity in dementia

**DOI:** 10.1093/ageing/afag235

**Published:** 2026-08-11

**Authors:** Shanshan Wang, Yang Fei, Yat Wa Justina Liu, Daphne Sze Ki Cheung, Sze Him Isaac Leung, Jiaming Xiong, Cypher Ho Au-Yeung, Wendy Moyle

**Affiliations:** School of Nursing, The Hong Kong Polytechnic University, Hong Kong SAR, China; School of Nursing, The Hong Kong Polytechnic University, Hong Kong SAR, China; School of Nursing, The Hong Kong Polytechnic University, Hong Kong SAR, China; School of Nursing and Midwifery, Deakin University, Melbourne, Victoria, Australia; Faculty of Health, Institute for Health Transformation/Alfred Partnership, Deakin University, Melbourne, Victoria, Australia; Department of Statistics and Data Science, The Chinese University of Hong Kong, Hong Kong SAR, China; School of Nursing, The Hong Kong Polytechnic University, Hong Kong SAR, China; School of Nursing, The Hong Kong Polytechnic University, Hong Kong SAR, China; School of Nursing and Midwifery, Griffith University, Brisbane, Queensland, Australia

**Keywords:** physical activity, dyad, dementia, caregiver, qualitative study, older people

## Abstract

**Introduction:**

Persons with dementia and their caregivers are more physically inactive than cognitively healthy peers or non-caregivers, yet little is known about their views on dyadic physical activities. This study explored the perceptions, facilitators, barriers and expectations related to dyadic physical activity among persons with dementia and their caregivers.

**Methods:**

An exploratory dyadic qualitative study was conducted with 24 community-dwelling dyads in Hong Kong SAR, China. Semi-structured dyadic interviews were analysed using inductive thematic analysis.

**Results:**

Four themes comprising 22 subthemes were identified. Dyads recognised mutual physical, psychological and relational benefits and valued activities that fostered shared enjoyment and relational resonance, though some questioned dyadic activity’s relevance. Engagement was facilitated by clear rewards, established habits, everyday collaborative activities, culturally meaningful incentives and strong social and professional support in a facilitative environment. Participation was hindered by limited shared understanding of physical activity, negative emotions in partner, mismatched fitness levels and activity rhythms, social withdrawal by the person with dementia, time pressures and conflicting family attitudes. Dyads expected interventions to yield tangible health and fitness gains, be tailored to both partners’ capacities and interests, provide appropriate challenge and ensure safety through explicit risk management strategies.

**Conclusion:**

Dyadic physical activity is not simply ‘doing it together’. Instead, interventions should be tailored, health-oriented and relationally attuned, with explicit consideration of the practical and emotional challenges faced by both dyad members. Psychoeducation regarding the significance of physical activity may need to extend beyond the dyadic members to the wider family to promote ‘doing it together’.

## Key Points

- Dyadic physical activity is not simply `doing it together'.
- Dyadic physical activity interventions should be tailored, health-oriented and relationally attuned.
- Dyadic physical activity interventions for dementia should address both practical and emotional challenges of the dyad.

## Introduction

Physical activity, any bodily movement by skeletal muscles that increases energy expenditure, is essential for health [1]. However, people living with dementia are typically inactive: They average 21.6% less physical activity and 8.9% more sedentary time than cognitively healthy older adults, spending approximately two-thirds of their day being sedentary [2]. This inactivity worsens physical function, accelerates dementia progression and reduces quality of life. Family caregivers face similar declines in physical activity; over two-thirds are physically inactive after taking on caregiving duties [3], and many report poorer health, becoming ‘invisible secondary patients’ [4]. These parallel declines in activity may suggest the mutual influence between people living with dementia and their caregivers.

This perspective is supported by the Dyadic Illness Management Theory, which posits that health behaviours and outcomes within a dyad are mutually interdependent [5]. As dementia progresses, people with dementia and caregiver function as an interconnected emotional and behavioural system; thus, supporting both individuals is essential to preserving family quality of life [6]. Physical activity, a low-cost and accessible lifestyle behaviour, can have potential to benefit both the person with dementia and their caregiver. Systematic reviews confirm strong interconnections between patients and caregiver well-being [7] and show that dyadic interventions can yield mutual benefits [8]. Yet implementing dyadic activity and altering inactive lifestyles remains highly challenging [9].

It is therefore important to understand the perspectives, facilitators, barriers and expectations of dementia–caregiver dyads regarding physical activities, to enhance the feasibility and long-term effectiveness of dyadic interventions. This insight is critical to co-design strategies that reflect both members’ lived experiences, needs and preferences and sustain engagement in physical activity over time. However, in-depth qualitative evidence from dyadic perspectives remains limited. A review [9] synthesised facilitators, motivators and barriers among people with dementia but largely ignored caregivers’ roles and dyadic dynamics; it included only seven small-sample studies, limiting generalisability. To date, just two dyadic interview studies have been found. One included only four dyads within a mixed sample [10], while the other interviewed 15 dyads but focused solely on facilitators, barriers and motivators [11]. No studies have explored these issues among Chinese dementia–caregiver dyads, despite China having the world’s largest dementia population. This gap is particularly important because culturally specific features, such as family-cantered caregiving, filial responsibility and caregiver self-sacrifice, shape how physical activity is perceived, prioritised and practiced within the dyad. This study bridged this gap by exploring perspectives, facilitators, barriers and expectations regarding dyadic physical activity among Chinese individuals with dementia and their informal caregivers. The findings can inform evidence-based interventions, policies and practices to enhance the impact of dyadic physical activity for dementia–caregiver dyads from this community.

## Materials and methods

### Aims

To explore the perceptions of dyadic physical activities among people with mild-to-moderate dementia and their informal caregivers from the Chinese community, and to identify the facilitators, barriers and expectations for these activities.

### Study design

We used an exploratory qualitative design with semi-structured dyadic interviews involving individuals with mild-to-moderate dementia and their primary informal caregivers. The study followed the Consolidated Criteria for Reporting Qualitative Research (COREQ) checklist [12]. This study informs Phase I of a larger clinical trial.

### Participants and recruitment

Dementia caregiving dyads were recruited from six community centres in Hong Kong using purposive sampling. Social workers referred potential participants to the research team, and a trained research assistant screened them. People with dementia were included if they: (a) had a clinical diagnosis of mild-to-moderate dementia (Global Deterioration Scale score from 4 to 6) [13], (b) lived in the community, (c) could communicate verbally and in writing, (d) could walk independently and (e) were physically inactive per WHO guidelines, i.e. insufficient physical activity to meet WHO’s physical activity recommendations [14]. Caregivers were included if they were the primary caregiver and had provided care for at least 6 months to ensure that they had sufficient caregiving experience and familiarity with the daily routines, physical activity patterns, needs and challenges of the person living with dementia. Those with cognitive impairment were excluded. Sample size was determined by thematic saturation [15].

### Data collection

Interviews were conducted from November 2024 to December 2025 by two trained research assistants, with 8 years’ (C.H.A.) and 4 years’ research experience (J.X.), respectively. Both interviewers completed formal training in qualitative methods and population-specific interviewing and conducted a mock interview beforehand to ensure consistency and compliance to the topic guide and study protocol. The first author reviewed all recordings for protocol adherence and quality. To accommodate participants’ needs and preferences, interviews took place in private, comfortable settings—community centres (*n* = eight dyads), participants’ homes (*n* = nine dyads), a university meeting room (*n* = one dyad) or via Zoom (*n* = six dyads). Before each session, interviewers introduced themselves explained the study’s purpose and procedures in plain language. Participants were asked to indicate their understanding of the study and willingness to participate. Informed consent was obtained from both members of each dyad, and written informed consent was obtained before the interviews. For participants with dementia, their assent and ongoing willingness to participate were confirmed before and during the interview. No signs of distress or unwillingness were observed. As the interviews involved non-sensitive questions, each dyad was interviewed jointly to explore shared and differing experiences. This approach was appropriate because physical activity in dementia is often relational, involving caregiver support with encouragement, planning, accompaniment, safety monitoring and decision-making. Joint interviews also allowed caregivers to support recall and communication when needed, while enabling people with dementia to express their own views in the presence of a familiar person.

Participants were interviewed using the topic guide in Table 1, supplemented with flexible probing (e.g. idiographic memory, descriptive, exploratory and clarifying probes) [16]. The topic guide was developed based on the research objectives, relevant literature on physical activity among people living with dementia and caregivers and discussions within the research team. It was also refined following mock interview practice. Interviews lasted 36–56 min, were audio-recorded with consent and transcribed verbatim by a research assistant. Each participant was assigned a unique participant ID that was unrelated to their personal information. Participant IDs were used throughout the interview and transcription process to ensure anonymity. The first author verified transcription accuracy by cross-checking against recordings. After 21 dyads, no new themes emerged; three additional dyads were interviewed for verification [17]. Data collection ended with 24 dyads.

**Table 1:** Topic guide for interviews

#1	Tell me about your physical activities in daily life. *Idiographic memory probe:* Think about a recent week, tell me about the times when you were moving around or being physically active, what do you do? *Clarifying probe:* physical activities can be walking, gardening, exercise or active housework. *Descriptive probe:* how often do you do these activities? How long each time?
#2	What do you think of doing physical activities together with your care partner? *Explanatory probe:* how do you feel about being active together? What do you see as the benefits or drawbacks of doing activities with your care partner?
#3	What factors do you think would facilitate you doing activities together? *Descriptive probe:* could you describe any practical things that would help make it easier for you and your care partner to do activities together (e.g. people, service, time, equipment, support)? *Explanatory probe:* why do you think those factors in particular would make it easier? How would having [factors you mentioned] change what you’re able or willing to do together?
#4	What are your barriers for doing physical activities together with your care partner? *Idiographic memory probe:* can you describe a recent time when you wanted to do something together but it didn’t happen? What got in the way? *Explanatory probe:* how do these barriers influence your motivation or willingness to do activities together?
#5	What are your expectations for doing physical activities with your care partner? *Descriptive probe:* what would you want if you are asked to do physical activities together?
*Clarifying probes were used throughout the interview as appropriate, e.g. when you say ‘I’m just around the house,’ what kinds of things are you actually doing?*	

### Data analysis

Data were analysed using Braun and Clarke’s [18] six-phase inductive thematic analysis: (1) familiarisation; (2) initial coding; (3) generating themes; (4) reviewing themes; (5) refining, defining and naming themes and (6) writing the report. MAXQDA 2024 software was used for data management and analysis. The second author led transcript reading, coding and preliminary theme development for all transcripts; the first author independently reviewed transcripts and the coding framework, refined themes and reclassified subthemes and codes. Weekly discussions between authors resolved coding and interpretive differences, with final consensus reached via team consultation. Dyadic-level coding was applied—both members of each pair were coded concurrently per question—to enable individual- and dyad-level analysis. Appendix S1 shows the coding tree as an illustrative overview of key coding stages. When participants with dementia provided limited data (*n* = 9; i.e. consistently brief responses), caregiver input was used to contextualise and interpret them. Interviewers sought confirmation from the person with dementia using both verbal and nonverbal indicators, including brief agreement or disagreement, elaboration, nodding and facial expressions, to ensure that the caregiver’s interpretation accurately reflected their perspectives. Field notes were kept for those cases.

### Ethics

Ethical approval was obtained from the Institutional Review Board of the corresponding author’s institution. Participants received clear, accessible information about the study in plain language both verbally and in writing, with adequate time to decide on participation. Informed consent and audio recording permission were obtained from all. Interviews and surveys were anonymised; participants were identified only by unique reference numbers. All data were stored in encrypted folders on the institution’s secure computer.

### Rigour and reflexivity

Methodological rigour and credibility were ensured through systematic data collection, transcription and analysis. Interviews were conducted by experienced psychology or geriatric nursing researchers trained to build trust with participants. Participant dyads varied by gender, dementia stage, caregiving duration, caregiver employment status and relationship type, broadening perspective authenticity. Study context and participant details supported transferability assessments. All raw data and coding decisions were fully traceable. Transcripts were produced by Cantonese-English bilingual staff and verified against original recordings. Analysts drew on deep local knowledge of dementia care in Hong Kong, repeatedly reviewed audio and text data, reflected on emerging interpretations and held regular discussions with the principal investigator to identify themes, compare findings and resolve discrepancies. These iterative processes enhanced traceability and contributed to the credibility and trustworthiness of the analytical outcomes.

## Results

### Characteristics of participants

Of the 24 dyads, caregivers averaged 65.04 years (SD = 9.86), while people with dementia averaged 80.54 years (SD = 8.19). The average caregiving duration was 5.86 years (SD = 4.59). Most caregivers were female (91.7%), and half were spouses. Half of the dyads engaged in daily physical activity together. Detailed participant characteristics are in Table 2.

**Table 2:** Characteristics of participants

Characteristics	Mean (standard deviation)/*n* (%)
Caregiver (*n* = 24)	
Age	65.04 (9.86)
Caregiving duration (years)	5.86 (4.59)
Gender	
Male	2 (8.3%)
Female	22 (91.7%)
Relationship with care recipient	
Spouse	12 (50%)
Daughter	10 (41.7%)
Daughter-in-law	1 (4.2%)
Sister	1 (4.2%)
Employment status	
Full-time	4 (16.7%)
Part-time	2 (8.3%)
Retired	15 (62.5%)
Unemployed	3 (12.5%)
Marital status	
Married	15 (62.5%)
Single/never married	7 (29.2%)
Divorced	2 (8.3%)
Education level	
Primary school	4 (16.7%)
Secondary school	13 (54.2%)
Associate degree/higher diploma	2 (8.3%)
Bachelor’s degree	4 (16.7%)
Master’s degree or higher	1 (4.2%)
People with dementia (*n* = 24)	
Age	80.54 (8.19)
Gender	
Male	12 (50%)
Female	12 (50%)
Stage of dementia as measured by the Global Deterioration Scale	
Mild dementia	3 (12.5%)
Moderate dementia	5 (20.8%)
Moderately severe	16 (66.7%)
Education level	
No formal education	2 (8.3%)
Primary school	8 (33.3%)
Secondary school	12 (50%)
Associate degree/higher diploma	2 (8.3%)
The caregiver and the person with dementia exercise together	
Yes	12 dyads (50%)
No	12 dyads (50%)

### Themes and subthemes

Themes and subthemes are presented in Table 3 and elaborated in the sections below.

**Table 3:** Themes and subthemes

Theme	Subtheme
Perceptions of dyadic physical activities	1. Mutual physical and mental benefits for both members of the dyad
	2. Building relational resonance through dyadic activities
	3. Perceived lack of value or relevance of dyadic activity
	4. Discrepancies in attitudes towards dyadic activity
Facilitators of dyadic physical activities	1. Perceived rewards and meaningful outcomes
	2. Established habits
	3. Use of everyday collaborative activities as a stepping-stone
	4. Enjoyable and engaging activity design
	5. Culturally meaningful rewards as catalysts for dyadic engagement
	6. Initiatives and encouragement from either member of the dyad
	7. Social and professional support
	8. Supportive context and environment
Barriers to dyadic physical activities	1. Limited shared awareness and understanding of physical activity
	2. Negative emotional responses to activity by either member of the dyad
	3. Active social withdrawal of the person with dementia
	4. Variation in physical fitness levels and activity rhythms between dyadic members
	5. Scheduling and time-constraint conflicts
	6. Conflicting family attitudes and overprotective interference
Expectations for dyadic physical activities	1. Health and fitness benefits
	2. Tailoring of activities to dyadic functional capabilities and interests
	3. Appropriate difficulty and intensity of activity
	4. Assurance of safety and risk management during dyadic exercise

#### Perceptions of dyadic activities

##### Mutual physical and mental benefits for both members of the  dyad

Dyadic physical activities were viewed to benefit both caregivers and people with dementia. Caregivers reported that exercising together supported their own health and leisure needs while motivating the person with dementia to be more active, thus fulfilling both self-care and caregiving goals. As one caregiver said, ‘I feel happy, because I can get sunshine and exercise, and I can also encourage him to join me. It’s self-care and caregiving’ (CG11). Though fewer people with dementia commented directly, those who did valued the companionship, conversation and shared enjoyment: ‘Doing exercise together is nice. We chat and laugh. It’s good for our health and mental wellbeing’ (P11).

##### Building relational resonance through dyadic activities

Relational resonance refers to the process through which individuals recognise, affirm and align with each other’s emotions, experiences or meaning, creating a sense of mutual understanding, sameness and validation within the relationship [19]. Participants described dyadic physical activity as fostering relational resonance through four interrelated processes: establishing shared goals, experiencing resonance and alignment through exercising together, prioritising enjoyment over perfection and viewing dyadic exercise as a safe space for positive interaction. Caregivers stressed that exercising together created shared purpose and alignment, even with imperfect or uneven movements and emphasised continuity and togetherness over technical accuracy.

Doing exercise together, well, there’s a shared, common goal, yes. (…). Because when you go to exercise together, it’s the same movement, the same activity, which creates alignment, right. Yeah, so whether you can do it perfectly or not doesn’t really matter, because we were doing it together anyway. I don’t expect it to be exactly correct, because he might not be able to keep up, he may not follow along completely, and I might not be able to guide him to do it perfectly at that moment either. But since we are doing it continuously, that sense of doing it together and the resonance (is what matters). (CG21)

##### Perceived lack of value or relevance of dyadic activity

For dyads with no history of shared leisure or family activities, dyadic exercise often seemed irrelevant. One caregiver noted that, given their long-standing focus on essential daily tasks, such activities felt unnecessary and unlikely to foster mutual growth or tangible benefits. So, their time together remained practical and task-oriented.

Actually, there wasn’t really any ‘growing up together’ process, so it’s hard for me to even imagine it (doing exercise together). We tend to do more practical things when we’re together. Apart from things that truly have to be handled together. Usually work-related tasks or things I have to accompany her to do. We don’t really have normal family activities at home. There hasn’t been that kind of individualized process, so we hardly ever play together. When you think about it, it’s quite strange. (CG1)

##### Discrepancies in attitudes towards dyadic activity

Discrepancies in perspectives between caregivers and people with dementia were also found in relation to dyadic activity. To support safety and ability, caregivers adapted activities, e.g. slowing walking pace, reducing intensity, staying within sight, taking frequent breaks and prioritising safety. As one caregiver explained: ‘Sometimes, I have to accommodate her situation, and I can’t get too far away from her’ (CG18). These adjustments highlight caregivers’ protective role and sense of responsibility.

In contrast, some people with dementia expressed a preference for exercising alone, perceiving dyadic or group-based exercise as restrictive or intrusive. For them, joint exercise evoked feelings of pressure or loss of autonomy, ‘I won’t do exercise with you (caregiver). It’s too annoying’ (P2). These discrepancies underscore tensions between caregiver intentions to support and adapt and care recipients’ desires for independence and autonomy in physical activity participation.

#### Facilitators of dyadic activities

This theme delineates the facilitators that supported dementia caregiving dyads to engage in dyadic activity, spanning both internal and external influences.

##### Perceived rewards and meaningful outcomes

All caregivers indicated their willingness to engage in dyadic activities when possible. However, they noted that people living with dementia were sometimes reluctant. Several caregivers reported that simple, concrete rewards increased willingness, especially for low-intrinsic-value activities. Structured rewards and clear outcomes thus serve as practical tools to initiate and sustain engagement. As one caregiver explained, “She really likes it when there’s a reward. For example, if there’s one dollar for doing a movement, she would think ‘Wow, there’s a benefit,’ then she might do it (exercise)” (CG4).

##### Established habits

For some dyads, long-standing shared exercise habits, like Baduanjin (a traditional Chinese exercise), running or football, helped sustain dyadic activity after a dementia diagnosis. These routines were often part of one or both partners’ lifelong exercise identities. One participant said ‘We do Baduanjin together regularly’ (CG8). Another noted: ‘In the morning, I run with him. (...). Because he’s exercised his whole life and still enjoys it’ (CG17).

##### Use of everyday collaborative activities as a stepping-stone

Dyadic activity was defined broadly as any shared physical activity, not just formal exercise. Caregivers described everyday tasks, such as cooking, washing dishes, setting the table and grocery shopping as natural opportunities for collaboration, involving coordinated movement and interaction. One caregiver explained, ‘We do (activities together). Uh, I cook and he washes the dishes. We go grocery shopping together. I choose items and he carries them’ (CG21).

##### Enjoyable and engaging activity design

A caregiver emphasised that activities involving new experiences were more engaging than repetitive or competitive sports. Exploring new experiences together allowed both members of the dyad to be active while sharing enjoyable experiences, without performance pressure or comparison. As a caregiver said, ‘Traveling is a situation where we can exercise together. He needs exercise, and I also need exercise, and we get to explore something together. The outcome from that is good, it’s a new thing’ (CG12).

##### Culturally meaningful rewards as catalysts for dyadic  engagement

Regional cultural practices in Hong Kong, such as going out for Cantonese yum cha, emerged as important motivators for walking and other physical activity. Some caregivers described using yum cha as a culturally meaningful ‘reward’ to encourage participation, ‘He likes yum cha and insists on finishing it before he’s willing to go for a walk’ (CG21). Given the popularity of other local practices such as hiking, activities that some dyads had enjoyed earlier in life, these data suggest that anchoring dyadic activity to culturally valued routines and treats may enhance uptake and adherence.

##### Initiatives and encouragement from either member of the dyad

Dyadic activity was facilitated when either partner actively initiated or encouraged participation. Caregivers most often prompted exercise: ‘Yes, I do that. I encourage him to do it (exercise) every day’ (CG11). Although some people with dementia requested opportunities to go out: “She might ask to go out. She’d say something like, ‘I want to go for a walk today,’ or ‘It’s too stuffy at home’” (CG18).

##### Social and professional support

Professional guidance on physical activity was widely welcomed, particularly when delivered face-to-face. Many participants preferred one-to-one instruction to ensure they learned movements correctly and could ask questions in real time. Group activities were preferred by some, where peer interaction and shared learning enhanced motivation.

Face to face. This is something that’s very much about feeling. We’re not that generation that can just watch something and learn. We want to be able to ask the instructor, ‘Am I doing this right?’ or ‘Which muscle am I using now?’ and get an answer right away. It needs that kind of interaction. (CG12)

If you have a real group, older adults think, ‘Others are playing; it must be good; then I’ll play too.’ They have that mentality. That’s why I say, in a group, having a ‘popular granny’ (champion) makes it much easier than any other kind of incentive. (CG1)

##### Supportive context and environment

The physical environment strongly influenced dyadic activity feasibility. Limited home space was a common barrier, e.g. ‘there’s no place here (home) to do exercise together’ (CG10), while accessible venues (e.g. community centres), appropriately designed programmes (e.g. music-accompanied, simple movements) and supportive social settings facilitated participation.

If I need to go somewhere else (other than home and the center), I first have to get her out, and only then can I talk about other things. Here at the center, there are people to accompany her. I think using the center to do exercise is okay. (CG1)

You could consider integrating music into the exercise. This might give them a familiar sense or nostalgia. (…). Stretching exercises, ‘chair dancing’—that would work. You have music in the background, the movements are very simple, and it gets her moving. (CG16)

#### Barriers to dyadic activities

This theme captures the main factors that hindered people with dementia and their caregivers from engaging in dyadic physical activities. These map onto six interrelated subthemes.

##### Limited shared awareness and understanding of physical activity

Some dyads experienced a barrier to joint participation because they lacked a shared understanding or intention to engage in exercise together. As one person with dementia explained: ‘We each do our own thing (exercise), doing it if we feel like it, not really making a deliberate plan to do it together’ (P10). A caregiver attributed this to lifelong independence: ‘Because perhaps we’ve had a strong sense of independence since we were children’ (CG1). In these dyads, exercising together was not part of their relational repertoire. Rather, physical activity was viewed as an individual and autonomous behaviour rather than a joint practice. Without explicit support to frame it as shared, dyadic exercise remained unlikely, even when both partners were independently active.

##### Negative emotional responses to activity by either member of  the dyad

Dyadic activity was closely linked to both partners’ emotional states: ‘If I’m not in good mood, I don’t want to do exercise together’ (P21). Activities involving competition or performance-oriented exercises were experienced as emotionally aversive because they created pressure, comparison or the risk of ‘losing’. Emotional discomfort in either partner acts as a key barrier, suggesting that dyadic formats emphasising shared enjoyment over performance may help reduce emotional barriers.

“If it’s ‘me versus you’ with that competitive mindset, one wins, one loses, someone ends up unhappy, like ‘Why did you play like that?’ But if we try something new together, like going diving or exploring somewhere. ‘Hey, look at this, wow, it’s like this here’. We would find that much more enjoyable” (CG12)

##### Active social withdrawal of the person with dementia

Marked social withdrawal in people with dementia significantly constrains opportunities for dyadic engagement. Caregivers reported that long-standing avoidance of exercise and social contact poses a key barrier to participation in dyadic activities.

If she were willing, I could do it (dyadic activity) with her. But she simply refuses to move. It feels impossible; I’ve stopped expecting it. Even when the social worker comes to deliver meals, I ask them to get her to exercise or go downstairs, but she is extremely unwilling. She doesn’t like socialising. (CG4)

##### Variation in physical fitness levels and activity rhythms between  dyadic members

Differences in physical capacity, coordination and pace between partners hindered dyadic activity. Some caregivers found the person’s cognitive and motor impairments so overwhelming during sessions that activities became supervision rather than shared exercise. In other cases, the caregiver had lower physical capacity than the person with dementia.

Watching her exercise makes me happy, and she enjoys it too, even if she can’t do it well. But if I do it with her at home, it’s so infuriating. She moves all over the place, with no sense of direction. When I go with her, I end up focusing on her and neglecting myself. (CG2)

##### Scheduling and time-constraint conflicts

Competing time constraints limited dyadic activity even when both partners were motivated. People with dementia often had ample free time, but caregivers faced work and other responsibilities, making synchronised participation difficult. Many caregivers were too exhausted by caregiving and work to add exercise, despite valuing it. Dyadic activity must therefore fit realistically into both partners’ daily routines and energy levels. As one caregiver said: ‘She has plenty of free time, but I’m not.’ (CG1). A person with dementia also added: ‘When she’s free, I’m not; when I’m free, she isn’t.’ (P1).

##### Conflicting family attitudes and overprotective interference

Competing family demands could directly disrupt dyadic activity. In some cases, other family members unintentionally undermined the primary caregiver’s attempts to exercise with the person with dementia. Over-protective comments and frequent interruptions reduced the caregiver’s confidence and the feasibility of the activity.

For instance, while I’m teaching my mom (with dementia) exercises, my dad will say, ‘Be careful, be careful!’ because the movement might bump into the table, or ‘Watch out for the vase!’. He constantly chimes in from the side. He also advises against trying anything too difficult. Basically, I’m just trying to teach her, yet he keeps interfering with us. (CG19)

#### Expectations for dyadic activities

When considering potential dyadic physical activities, dyads focused on activity difficulty, type and intensity, expected health effects, individual adaptability and safety.

##### Health and fitness benefits

Dyads expected joint activities to deliver health benefits, particularly in maintaining or improving physical fitness and muscle strength. Caregivers emphasised preserving leg and core strength to prevent falls, maintain mobility and protect overall quality of life for both partners. Accordingly, they sought activities viewed as meaningful for functional preservation rather than participation for its own sake.

I’m fifty now, so I believe I need some muscle-building exercise too. She (person with dementia) really needs muscle training, especially for her waist and lower body. Her legs are in poor condition, and if her mobility improved, her quality of life would be much better. Even if her health doesn’t improve dramatically, at least if her legs were stronger, I wouldn’t worry so much about her falling when she gets up at night to go to the bathroom. (CG19)

##### Tailoring of activities to dyadic functional capabilities and  interests

Participants repeatedly emphasised that the acceptability of any dyadic programme depended on its fit with both partners’ functional capabilities and preferences. Physical issues in either member (e.g. pain, instability, limited stamina) could restrict feasible exercises, making individualised tailoring essential. As one caregiver explained: ‘Because his legs aren’t very steady when standing. I believe exercises designed to be done while sitting would be a better option’ (CG11).

##### Appropriate difficulty and intensity of activity

An appropriate level of difficulty was considered fundamental. ‘Appropriate’ did not mean overly simple; rather, exercises needed to be meaningfully challenging, but not so demanding that either partner felt frustrated or exhausted. If activities felt childish or overly demanding, dyads may lose motivation. As one caregiver stated,

‘I took her (person with dementia) to the community center, but I couldn’t stand it—the whole group included people in severe condition, and the activities were things my grandson would play with. So, I’d rather wait outside’ (CG2). A person with dementia similarly noted, ‘Maybe do some gentler exercises that better match my ability’ (P11).

##### Assurance of safety and risk management during dyadic exercise

Safety was viewed as a non-negotiable foundation for any dyadic physical activity. Caregivers were acutely aware of risks associated with cognitive impairment, balance problems and unrecognised injuries, and therefore, expected programmes to proactively address risk:

‘It really has to be safety first. Because even if I supervise him 24 hours, bad things still happen. Sometimes his skin just gets all red for no apparent reason. Ask him where he bumped himself, and he can’t tell me. So, safety is really essential’ (CG6).

## Discussion

This is among the first studies to explore perceptions, facilitators, barriers and expectations around physical activity from the perspective of people with dementia and their family caregivers—as dyads—and the first in a Chinese context. By examining both dyad members together, it yields novel insights to guide the design of dyadic physical activity programmes that support the health and well-being of both groups. Many of the identified subthemes align with the PHYT-in-dementia framework [20], which conceptualises physical activity participation in dementia as shaped by dynamic interactions among the person living with dementia, the social environment, the physical environment and the characteristics of the activity itself. In particular, our findings reinforce the framework’s core proposition: participation is not driven solely by personal factors, such as individual motivation or health knowledge, but emerges from a dynamic, interdependent system encompassing personal, relational, environmental and sociocultural factors.

Consistent with previous research, participants recognised mutual physical and psychological benefits of shared physical activity [11]. This study extends that evidence by identifying ‘relational resonance’, a core benefit reported by dyads. They described joint activity as fostering togetherness, mutual appreciation and shared joy, moments that helped sustain connection and relationship continuity amid dementia-related cognitive decline [21]. This suggests that dyadic physical activity functions not only as a health promotion strategy but also as a relationship-supportive intervention. However, key challenges emerged. Some dyads experienced misalignments in attitudes, especially when functional capacities differed markedly, or when the person with dementia perceived the caregiver’s involvement as pressuring or controlling. These tensions highlight the need for shared decision-making and individually tailored activities. Additionally, dyads without a history of shared family activities questioned the value of dyadic exercise altogether. This may be influenced by cultural norms of family-cantered collectivism in Chinese communities, where caregivers may feel expected to prioritise the needs of the person living with dementia and sacrifice their own time and energy. Spending time on activities other than caregiving may be perceived as inconsistent with expected family obligations [22]. Since social integration and family interaction support cognitive and social health in older adults, addressing such scepticism is a critical first step in designing effective interventions [23].

This study also advances knowledge by highlighting the significance of integrating physical activities into the lived realities, preferences and cultural context of people with dementia and their caregivers. Building on evidence about perceived rewards, health benefits and social support [11], it reveals those everyday collaborative activities (e.g. like cooking, walking to the market or doing chores) serve as potential entry points. Framing activity as ‘doing daily life together,’ rather than formal exercise reduces resistance, likely because it lowers cognitive demand, maintains a sense of competence and avoids the stigma of ‘rehabilitation’ [24]. Enjoyable and engaging design rooted in fun, novelty and shared joy also emerged as a key facilitator, suggesting intrinsic motivation sustains participation better than health-focused goals alone [25]. Culturally meaningful rewards, such as sharing traditional meals or practicing familiar rituals after activity, strengthen adherence by linking movement to identity and social roles. Finally, established routines and prior shared hobbies supported engagement, likely by leveraging preserved procedural memory in early-stage dementia [26]. Collectively, these insights underscore that effective dyadic interventions should be habit-based, culturally grounded and embedded in real-life contexts.

Barriers to dyadic physical activity reflected multi-level constraints that mirror, and sometimes counteract, the perceptions and facilitators reported by participants. Besides the variation in physical fitness levels and activity rhythms between dyadic members, along with the scheduling problems identified by previous research [11], the findings of this study highlight the importance of shared awareness and understanding of physical activity within dyads and the wider family. Although participants recognised it’s health and relational benefits, few viewed it as an important shared endeavour. This may be related to the Chinese cultural norm of family-cantered collectivism and filial piety, which shapes caregivers to prioritise supervision and safety over physical activity [27]. This weakened its translation into everyday practice and diminished the perceived rewards and motivation. These findings underscore the need for psychoeducation targeting both dyad members as a core element of intervention design.

This study also identified that dyadic activity is not limited to the dyad but extends to the wider family. Family-system barriers, such as conflicting attitudes and overprotective interference, emerged as a novel, previously unreported barrier. Though well-intentioned, family members’ safety concerns, doubts about activity relevance and cautionary remarks can disrupt dyadic engagement, undermine caregiver confidence, intensify negative emotional responses and reinforce sedentary norms [28]. Effective interventions should therefore expand beyond the dyad to include family-level education and consensus-building on safety management, the relational value of shared activity and acceptable risk levels. Communication training and family-conflict resolution could be integrated into dyadic physical activity programmes. Another new finding was active social withdrawal by people with dementia, an internal avoidance of social and physical interaction, even when encouraged by caregivers, this may be linked to apathy or passivity symptoms [29]. This withdrawal directly limits opportunities for relational resonance through dyadic activity, warranting targeted strategies to re-engage individuals socially and physically.

Expectations for dyadic physical activities underscored the central role of perceived health benefits in motivating participation and the need to tailor activities to each dyad’s functional abilities and interests. Participants expected tangible health and fitness benefits, not just a recreational addition, aligning with research showing that caregivers and people with dementia prioritise activities that justify their time and effort [30]. This may be particularly important for caregivers, who often invest additional time, energy and resources to support the person with dementia on top of routine care duties. Therefore, dyadic physical activity interventions may be more acceptable when they clearly articulate benefits and minimise additional burden on caregivers. Throughout the interviews, participants stressed that no ‘one-size-fits-all’ activity exists: difficulty and intensity must be adjusted to individual capabilities to ensure safety and support motivation, confidence and sustained engagement. Overall, these findings suggest that dyadic programmes must go beyond simply ‘doing it together’ by making health benefits and limitations explicit, accommodating dementia-related physical, emotional and social challenges and embedding strong safety and support measures.

### Limitations

This study has several limitations. First, purposive sampling via community centres may limit representativeness, as findings may not reflect perspectives of individuals not engaged with services or reluctant to discuss physical activity. Second, cognitive and verbal impairments among people with dementia may have affected their ability to understand questions or express experiences fully, even with caregiver support and careful participant selection, the possibility for missed or misrepresented information cannot be diminished. Although caregiver input helped contextualise the accounts of people with dementia who showed apathy or limited engagement and verbal and non-verbal confirmation were used, this may have affected the representation of the person with dementia’s own perspective. In addition, although a joint interview approach was appropriate for exploring physical activity as a shared and relational experience, it may have limited disclosure. Socially desirable answers, deference by the person with dementia towards the caregiver or suppression of divergent views may have occurred. Besides, six interviews were conducted online due to travel constraints; although field notes were kept and dyad reactions observed, the virtual format may have reduced the depth and richness of behavioural observation compared to in-person interviews. Last, because most caregivers were female and half were spousal caregivers, the findings may be less transferable to dyads involving male caregivers or alternative caregiving arrangements.

## Conclusion

The findings highlight the need for tailored, health-focused physical activities that are grounded in the practical and emotional realities of people living with dementia and their informal caregivers. To promote ‘doing it together,’ psychoeducation about the importance of physical activity may also be required and extend beyond the dyad to the wider family.

## Supplementary Material

Supplementary_materials_afag235

## Data Availability

The data is not publicly available due to privacy and ethical restrictions. The data that support the findings of this study are available from the corresponding author upon reasonable request.
