# Supplementary material for: ‘Doing it together?’ A dyadic qualitative study of perceptions, facilitators, barriers and expectations for physical activity in dementia
Source: Age Ageing. 2026 Aug 11;55(8):afag235. doi: 10.1093/ageing/afag235 (PMC13461214; doi:10.1093/ageing/afag235)
Supplement: Supplementary file 1 [file supplementary_materials_afag235.docx]

**“Doing It Together?” A Dyadic Qualitative Study of Perceptions, Facilitators, Barriers, and Expectations for Physical Activity in Dementia**

**Appendix 1.** Example of the coding tree

| **Raw transcription** | **Meaning bearing content** | **Code** | **Subtheme** | **Theme** |
| --- | --- | --- | --- | --- |
| P11: “Doing exercise together is nice. When the two people do it, we can chat and laugh during the process.” | enjoy the process | enjoy dyadic exercise: fun and pleasant | Mutual physical and psychological benefits for both members of the dyad | Perceptions |
| CG4: “(...) She really likes it when there is a reward. For example, if there’s one dollar for doing a movement, she would think ‘Wow, there’s a benefit.’ then she might do it (exercise). (...). It’s not that she wants to get some so-called benefits, and it is just that if she does something and gets a little reward, then she will be willing to do it. …” | one dollar for doing a movement; does something and gets a little reward | rewards or encouragement for activity | Perceived rewards and meaningful outcomes | Facilitators |
| P1: “When she’s free, I’m not; when I’m free, she (the caregiver) isn’t.” | She is free while I’m not free. | difficulty in finding mutually available time for exercise | Scheduling and time‑constraint conflicts | Barriers |
| CG6: “Then it probably really has to be safety first. Because even if I supervise him 24 hours, bad things still happen … Ask him where he bumped himself, and he can’t tell me. So, safety is really essential.” | Be safe first; Safety is really essential.” | emphasize the safety during activity | Assurance of safety and risk management during dyadic exercise | Expectations |

*Notes:* P denotes people with dementia and CG denotes the caregiver.
